# A perspective on AI implementation in medical imaging in LMICs: challenges, priorities, and strategies

**DOI:** 10.1007/s00330-025-12031-z

**Published:** 2025-10-23

**Authors:** Ahmed Marey, Ona Ambrozaite, Ahmed Afifi, Ritu Agarwal, Rama Chellappa, Sola Adeleke, Muhammad Umair

**Affiliations:** 1 Department of Radiology, Sheikh Khalifa Medical City, AbuDhabi, United Arab Emirates; 2 Department of Chemistry at the Krieger School of Arts & Sciences, Johns Hopkins University, Baltimore, MD USA; 3 Benha University Faculty of Medicine, Banha, Egypt; 4 Johns Hopkins Carey Business School, Baltimore, MD USA; 5 Department of Electrical and Computer Engineering, The Johns Hopkins University, Baltimore, MD USA; 6 School of Biomedical Engineering & Imaging Sciences, King’s College London, London, UK; 7 Russell H. Morgan Department of Radiology and Radiological Sciences, The Johns Hopkins University, Baltimore, MD USA

**Keywords:** Artificial intelligence, Medical imaging, Low- and middle-income countries (LMICs), Health policy, Radiology implementation

## Abstract

**Objectives:**

Artificial intelligence (AI) promises to accelerate and democratize medical imaging, yet low- and middle-income countries (LMICs) face distinct barriers to adoption. This perspective identifies those barriers and proposes an action-oriented roadmap.

**Materials and methods:**

Insights were synthesized from a Johns Hopkins Science Diplomacy Hub workshop (18 experts in radiology, AI, and health policy) and a scoping review of peer-reviewed and grey literature. Workshop discussions were transcribed, thematically coded, and iteratively validated to reach consensus.

**Results:**

Five interlocking barriers were prioritized: (1) infrastructure gaps—scarce imaging devices, unstable power, and limited bandwidth; (2) data deficiencies—small, non-representative, or ethically constrained datasets; (3) workforce shortages and brain drain; (4) uncertain ethical, regulatory, and medicolegal frameworks; and (5) financing and sustainability constraints. Case studies from Nigeria, Uganda, and Colombia showed that low-field MRI, cloud-based PACS, community-engaged data collection, and public–private partnerships can successfully mitigate several of these challenges.

**Conclusions:**

Targeted policy levers—including shared procurement of low-cost hardware, regional AI and data hubs, train-the-trainer workforce programs, and harmonized regulation—can enable LMIC health systems to deploy AI imaging responsibly, shorten diagnostic delays, and improve patient outcomes. Lessons are transferable to resource-constrained settings worldwide.

**Key Points:**

***Question*** *How can LMICs overcome infrastructure, data, workforce, regulatory, and financing barriers to implement artificial-intelligence tools in clinical medical imaging*?

***Findings*** *Our multinational consensus identifies five obstacles and maps each to actionable levers: low-cost hardware, regional data hubs, train-the-trainer schemes, harmonized regulation, blended financing*.

***Clinical relevance*** *Implementing these targeted measures enables LMIC health systems to deploy AI imaging reliably, shorten diagnostic delays, and improve patient outcomes while reducing dependence on external expertise*.

## Introduction

**Fig. 1:**
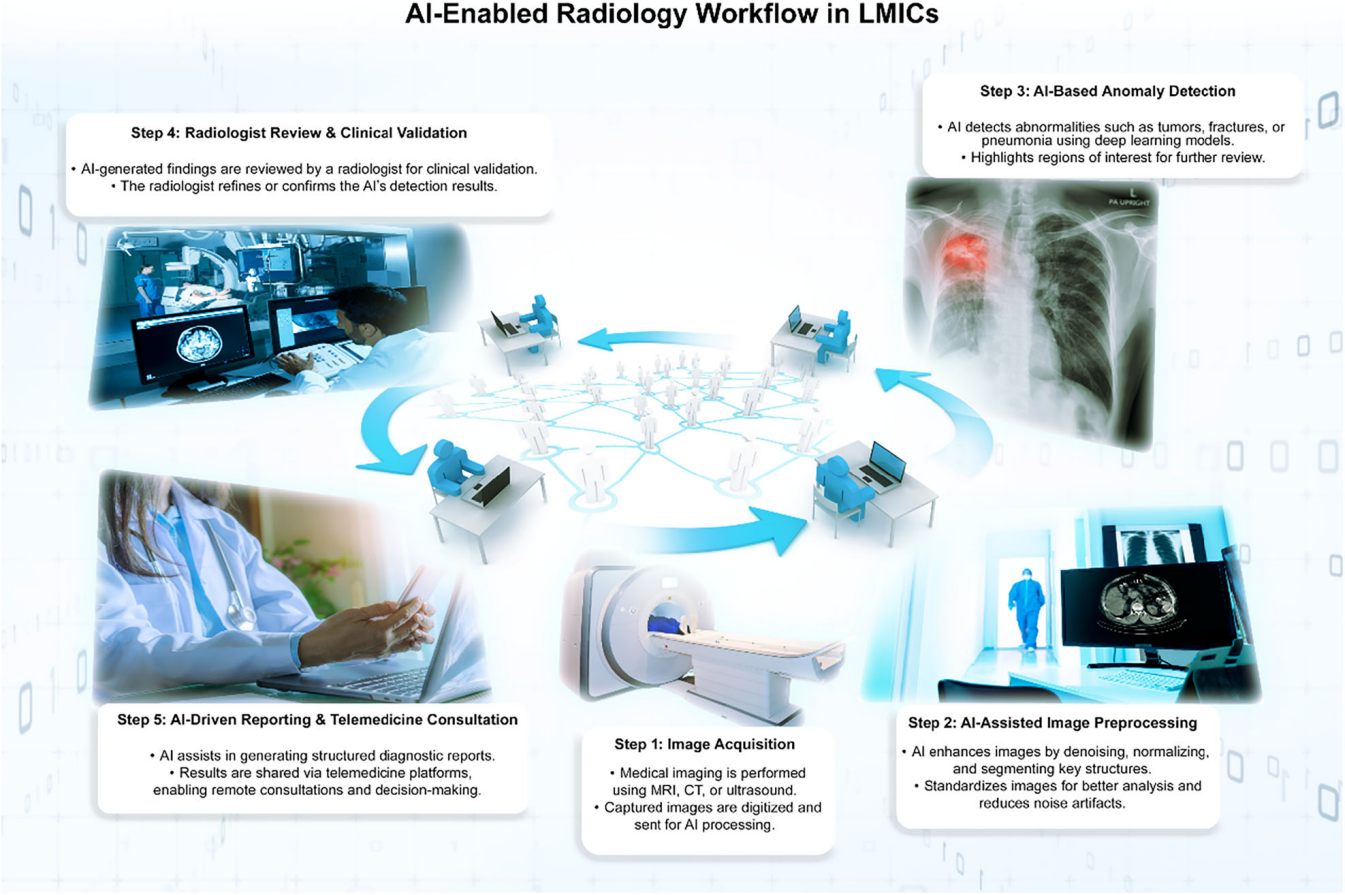
AI implementation roadmap for LMICs

Medical imaging technologies, such as magnetic resonance imaging (MRI), computed tomography (CT), and ultrasound, have long served as cornerstones of modern healthcare by enabling clinicians to diagnose disease with greater accuracy and speed. Today, artificial intelligence (AI) is ushering in a new era of innovation in medical imaging, offering a broad spectrum of capabilities that range from automating routine image analysis to assisting physicians in complex diagnostic evaluations. By processing vast quantities of imaging data and detecting subtle patterns beyond the scope of human observation, AI-driven tools have the potential to significantly improve both the accuracy of diagnoses and the timeliness of treatment (Fig. 1) [1–5].

Artificial-intelligence rollouts do not occur in a political vacuum. AI can entrench existing power asymmetries in data ownership, model development, and benefit sharing unless equity and justice are made explicit design goals [6, 7]. Khan et al call this emerging threat “AI colonialism”, urging co-creation with communities that historically supplied data but captured little value [8, 9]. We therefore frame the present roadmap as a contribution to this wider discourse while remaining action-oriented for implementers.

However, despite these advantages, low- and middle-income countries (LMICs) often struggle with basic healthcare infrastructure, which can hamper the successful adoption of AI. Many of these regions face persistent challenges such as unstable electricity and inadequate internet connectivity—components that are critical for running AI-powered solutions [10, 11]. Furthermore, the absence of reliable, context-relevant datasets trained on local patient populations undermines the accuracy and clinical relevance of AI models developed primarily in high-income countries (HICs) [12, 13]. Insufficient training opportunities for healthcare professionals and the lack of a robust cadre of local AI experts exacerbate these limitations, creating barriers to both effective implementation and long-term sustainability [14–17]. Considerable challenges notwithstanding, growing data highlights the capacity of AI to alleviate challenges to radiology and medical practices in LMICs [18, 19].

In response to these challenges, researchers and physicians from leading institutions like Johns Hopkins University and King’s College London, among others, joined forces during the Science Diplomacy Hub’s “Challenges of AI Implementation in Medical Imaging in LMIC Settings” event. Experts from multiple disciplines, including radiology, data science/AI, business, public health, and policy, provided comprehensive insights that form the basis of this expert opinion. By consolidating their expert viewpoints, this document identifies the most pressing hurdles limiting the integration of AI-driven medical imaging in LMICs, highlights priority areas for intervention, and proposes strategic recommendations for achieving scalable, equitable, and ethically grounded AI implementation. Through collaboration and targeted investment, it is hoped that these recommendations will serve as a roadmap for stakeholders—ranging from healthcare providers and policymakers to technology developers and international funders—committed to strengthening diagnostic capabilities and improving patient outcomes in resource-limited settings [20–23].

## Methods and perspective-building approach

### Preparatory literature review

Prior to the workshop, we conducted a scoping review of peer-reviewed articles, institutional reports, and policy documents related to AI in LMIC medical imaging.

We searched PubMed, Scopus, and IEEE Xplore from inception to 31 January 2025 using (“medical imaging” AND “artificial intelligence” AND [World Bank LMIC country list]) AND (“implementation” OR “deployment” OR “real-world” OR “workflow”). After de-duplication in EndNote, two reviewers (A.M., M.U.) independently screened titles/abstracts; disagreements were resolved by consensus. Full texts meeting all the following criteria were included: (i) human-subject imaging study performed in situ in an LMIC; (ii) describes an AI tool already deployed or undergoing prospective clinical validation; (iii) reports at least one implementation outcome (e.g., accuracy, turnaround time, cost, and acceptability). Exclusion criteria: purely technical algorithm papers without patient data, animal studies, editorials, and reviews.

This review examined key themes including infrastructure barriers, data privacy and ethics, workforce capacity, and financing models—thereby guiding the development of a semi-structured discussion guide for workshop sessions.

### Workshop composition and structure

The consensus presented in this manuscript was derived from a single, 2-h workshop entitled *“Challenges of AI Implementation in Medical Imaging in LMIC Settings,”* hosted by the Johns Hopkins Science Diplomacy Hub on 6 February 2025 (16:00—18:00 EST) at the Johns Hopkins Bloomberg Center, 555 Pennsylvania Avenue NW, Washington, DC (Room 822).

- 16:10—Welcome

- Opening remarks by Ona Ambrozaite, Co-Director, Johns Hopkins University Science Diplomacy Hub.

- 16:15–6:55—Flash talks (10 min each):
  - Ritu Agarwal – Digital health & AI adoption.
  - Rama Chellappa—technical frontiers in medical image AI.
  - Sola Adeleke—oncology imaging needs in LMICs.
  - Muhammad Umair—radiology workflow integration.

- 16:55–17:30—Panel discussion

- Moderated dialog on implementation barriers, priority actions, and the role of international partnerships.

- 17:30–18:00—Networking

- Informal breakout conversations over refreshments.

### Participant profile

- Speakers (*n* = 4): senior scholars and clinicians with peer-reviewed portfolios in AI or LMIC radiology.
- Audience (~40 attendees): scientists, diplomats, graduate students, and private-sector representatives from eight countries, providing a multidisciplinary and multi-regional perspective.

### Workshop limitations

First, the workshop had only four expert speakers. In addition, although the 40-person audience spanned eight countries, only 22 (55%) contributed vocally to the consensus exercise. Professional representation skewed toward academic radiologists, potentially under-representing primary-care clinicians and patients. We mitigated dominance by:

1. Structured agenda (flash talks → panel) ensured equal speaking time for each expert.
2. Rotating moderators and time-keeping prompts prevented dominance by any single voice.
3. Anonymous Slido polling captured audience priorities and questions in real time, allowing quieter participants—including LMIC-based attendees joining virtually—to influence the discussion.
4. Two independent note-takers documented proceedings for subsequent thematic coding (see the coming section).

These design features promoted balanced participation and enriched the dataset used for the thematic analysis that follows. Nevertheless, selection bias and social desirability bias remain possible.

### Data collection and thematic analysis

Two independent note-takers documented each roundtable and breakout discussion, generating textual data that was then uploaded into MAXQDA, a qualitative analysis software. Researchers applied a grounded theory approach to code these notes, iteratively identifying major themes (e.g., “infrastructure gaps,” “ethical data usage,” “training deficits,” and “financing challenges”) and sub-themes that offered more nuanced or context-specific viewpoints.

- Theme selection criteria: codes that appeared in ≥ 70% of the transcripts were elevated to major themes; codes that emerged in more context-specific or unique circumstances were deemed sub-themes and further discussed for relevance to the final recommendations.

### Perspective-building and validation

After initial coding, a preliminary perspective was drafted and circulated among workshop speakers. Recommendations were obtained from them on the preliminary perspective. These suggestions were discussed via email exchanges to incorporate all viewpoints. Final refinements were made based on these discussions, and a revised consensus statement was then approved by all participants.

**Fig. 2:**
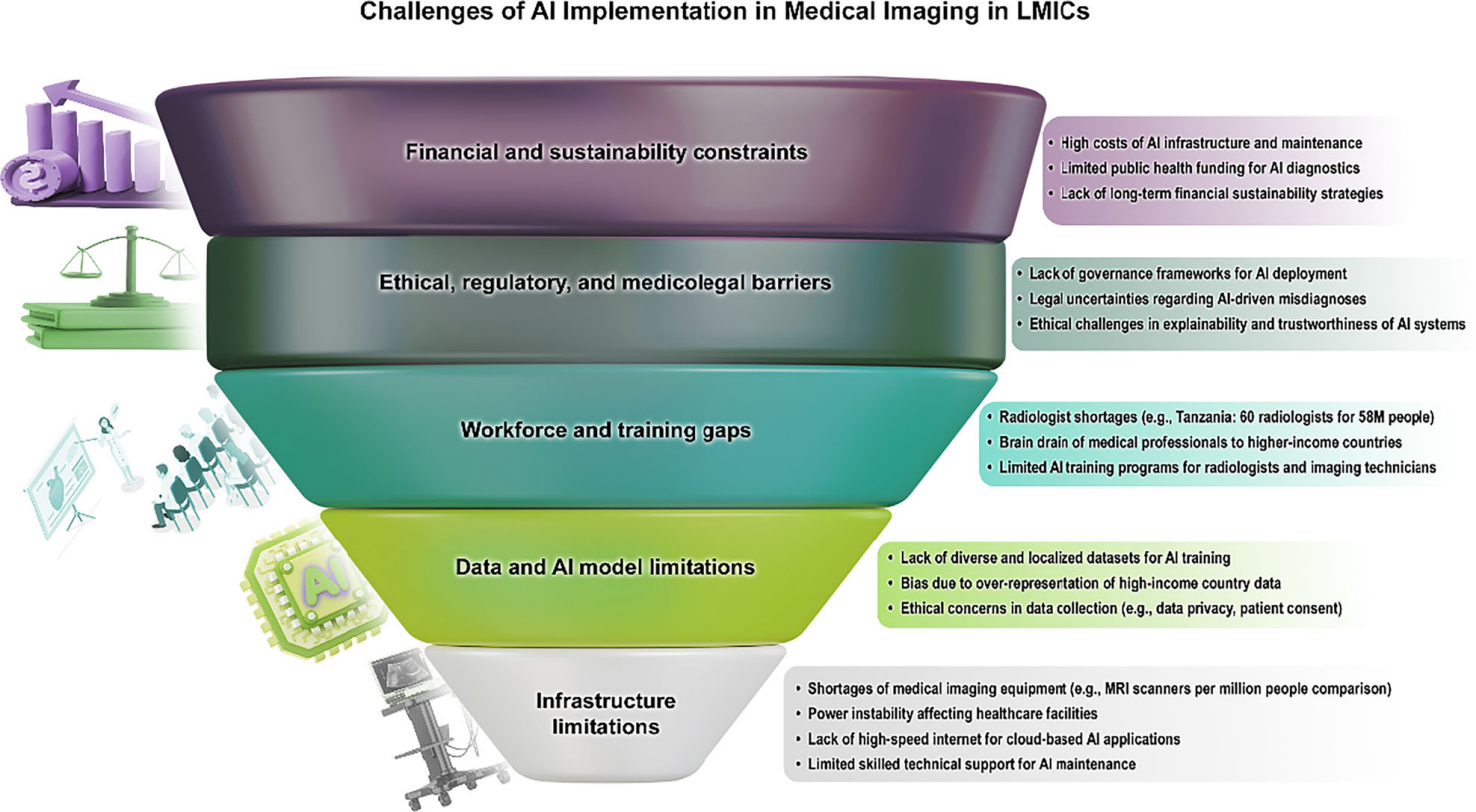
Challenges of AI implementation in medical imaging in LMICs

## Key challenges identified (Fig. 2)

### Infrastructure limitations

A foundational barrier to AI-driven medical imaging in LMICs is the shortage of essential diagnostic equipment. While HICs usually have multiple MRI and CT scanners available across different healthcare settings, many LMICs struggle to obtain even a single unit [24, 25]. In 2021, Sub-Saharan Africa, for instance, averages only 0.3 MRI units per million people, with 11 nations in the region having no MRI scanners at all [20]. This stark reality not only restricts the ability of healthcare providers to diagnose diseases accurately but also limits the volume and diversity of imaging data required to train and validate AI models effectively [26, 27].

In addition to equipment shortages, the absence of stable healthcare infrastructure—such as reliable electricity and modern imaging hardware—further impedes the deployment of AI-driven systems [27, 28]. These limitations inflate the cost and complexity of procuring and maintaining essential components, making them inaccessible to many resource-constrained settings. Even in sites equipped with basic imaging technology, the lack of connectivity—whether due to weak internet service or frequent power outages—often renders cloud-based AI platforms impractical [29]. Beyond this, the scarcity of skilled technical support raises concerns about the long-term sustainability of AI systems, as ongoing maintenance and troubleshooting cannot be guaranteed in many LMIC contexts [9].

Despite these formidable obstacles, innovative initiatives have demonstrated the feasibility of bridging infrastructural gaps. RAD-AID International, for example, has partnered with industry leaders and academic institutions to enhance radiology services and AI capabilities in LMICs [30]. A noteworthy case is the University College Hospital in Ibadan, Nigeria, where RAD-AID established a hybrid cloud-based picture archiving and communication system (PACS) that both modernized radiology services and incorporated locally validated AI software for chest radiograph interpretation [31]. Crucially, it was integrated in a way that respected local workflows, underlining that context-specific solutions—coupled with capacity-building and ongoing support—can transform AI from an unattainable aspiration into a tangible reality.

RAD-AID’s decade-long, site-level experience shows that AI adoption succeeds when education and infrastructure roll-out occur together [17]. Our roadmap shifts those ground-truth lessons upstream to the policy layer—addressing reimbursement, legal liability, and data sovereignty, all of which ultimately shape RAD-AID’s projects.

We therefore propose a field-to-policy feedback loop: (i) subject to future collaboration, RAD-AID could pilot our Digital Sovereignty Compact and monitoring dashboard at three of its existing partner hospitals (Ghana, Nepal, Peru) during 2025–2026, and (ii) the resulting outcome data would refine the Compact before potential regional legislative uptake. Such a partnership, if pursued, would avoid duplication and amplify collective impact.

### Data and AI model limitations

A recurring obstacle is the sheer discrepancy in scale between the data on which AI models are trained and the complexity of real-world clinical practice. Even rigorously validated models may fall short when deployed on large patient populations. For example, a model with 99% sensitivity could still miss thousands of cases out of a million scans, highlighting the pressing need for ongoing, post-deployment performance evaluations. Recent research by Ahluwalia et al (2023) illustrates this point: the team found significant drops in a model’s sensitivity in younger patients—up to 33%—likely due to the underrepresentation of that demographic in the training data [32]. This underscores a broader concern about uncritically deploying HIC-trained models in LMIC contexts, where disease prevalence, demographics, and healthcare practices diverge markedly from the high-income settings in which these models were developed [12, 13, 33].

Moreover, the ethical sourcing of training data remains a fundamental issue. Of particular concern is a potential shortage of training data for marginalized populations that can introduce algorithmic bias into the AI models, affecting the accuracy of model performance [34, 35].

Projects like the ScanNav FetalCheck initiative in Uganda show that AI can be integrated responsibly when data acquisition is transparent, culturally sensitive, and well-aligned with local healthcare needs [36]. In Uganda—where specialized prenatal care is often limited—AI-assisted ultrasound technology enabled early detection of complications by accurately dating pregnancies without requiring specialist sonographers. Crucially, the project embraced informed consent, robust anonymization protocols, and government collaboration, demonstrating that AI can be introduced with respect for both patient autonomy and community trust [37].

Another illustrative case is the Segment Anything Model with Bounding-box-guided Prompts and an ensemble voting network (SAMBA) approach for glioma segmentation in sub-Saharan Africa [38]. By leveraging the BraTS-Africa dataset—tailored specifically for local imaging constraints—researchers achieved high segmentation accuracy despite having limited data (around 60 cases) [39–41]. This fine-tuning of a general-purpose AI model (SAM) highlights how localizing large, pre-trained systems can bridge the gap between universal AI advances and the specific realities of LMIC healthcare. Projects like the Africa Neuroimaging Archive (AfNiA) further support this mission by creating publicly accessible brain MRI repositories, thus promoting population-specific data acquisition and FAIR (findability, accessibility, interoperability, and reusability) data practices [42].

Collectively, these examples illustrate why context-specific data collection and validation protocols should be integral to AI development in LMICs: without careful attention to demographic underrepresentation, ethical data-sharing, and cultural feasibility, well-intentioned AI projects risk perpetuating health inequities rather than alleviating them [9–11, 43].

Two basic questions that arise in building AI models are: Will they work everywhere, and will they work for everyone. The first question deals with challenges due to data shift due to differences in probability distributions between training and test data, and the second question deals with whether the AI models exhibit any bias towards subgroups of the population. Many methods [44] for domain adaptation and generalization are being developed; recently, adversarial training [45] and knowledge distillation methods [46] have been developed for bias detection and mitigation.

Instead of pursuing a universal ‘one-size-fits-all’ model, some recommend a hub-and-spoke fine-tuning pathway: a publicly released foundation model is first adapted to local data via parameter-efficient federated learning, then re-validated quarterly using surveillance dashboards that flag performance drift [47]. This approach balances global knowledge-sharing with context-specific optimization.

### Workforce and training gaps

Another critical challenge involves the severe shortage of qualified radiologists and AI specialists in resource-limited settings. For instance, Tanzania has only 60 radiologists serving a population of over 58 million people, while Pakistan faces a similar scarcity with just one radiologist per 500,000 inhabitants [16]. Such constraints not only limit patient access to expert diagnostic services but also burden existing clinicians with an unsustainable workload, often leading to delayed care and missed diagnoses [48, 49].

Moreover, the phenomenon of brain drain—where highly trained professionals migrate to developed countries for better opportunities—further exacerbates workforce gaps [50, 51]. This can leave already overstretched healthcare systems in LMICs even more vulnerable. While AI-driven tools offer significant potential to streamline workflows and improve diagnostic accuracy, they are not substitutes for human expertise. Instead, AI should be viewed as a complement that empowers radiologists and clinicians to deliver better care, provided we invest in building and retaining a robust local workforce [52]. The “human-in-the-loop” approach involves continuous oversight by a healthcare professional working alongside an AI tool, ensuring that clinical judgment and computational insights complement one another for improved safety and accuracy [53].

In this vein, programs like scan with me (SWiM), developed by the Consortium for Advancement of MRI Education and Research in Africa (CAMERA), illustrate how targeted capacity-building can address both workforce shortages and ethical imperatives [54]. By providing a free, comprehensive curriculum to MRI technologists in Africa, Latin America, and Asia, SWiM equips participants with the skills needed for advanced image analysis and clinical care—particularly in high-burden areas like cardiac MRI (CMR) [55]. Crucially, it not only boosts MRI accessibility but also fosters local innovation, allowing technologists to adapt AI solutions to their unique clinical realities [56].

Another notable initiative, CONNExIN (the COmprehensive Neuroimaging aNalysis Experience In resource-constrained settings program), focuses on advanced neuroimaging training in LMICs [57]. Like SWiM, it follows a Teach-Try-Use strategy that combines case-based learning, observerships, and real-world application. By empowering local researchers and clinicians to tackle pressing neuroscience challenges, CONNExIN helps mitigate the risks of talent outflow and ensures LMICs are active contributors to scientific and clinical advancements [58].

Taken together, these examples demonstrate that robust workforce development—anchored by local training, ethical engagement, and practical incentives—is essential for AI-enabled healthcare to succeed. Without such initiatives, even the best-designed AI tools risk exacerbating health inequities if there are not enough in-country professionals to operate, maintain, and continually refine these systems.

### Ethical, regulatory, and medicolegal barriers

Widespread adoption of AI in medical imaging also raises pressing ethical and legal considerations. Many existing AI models operate as “black boxes,” making it difficult for clinicians to interpret how specific algorithms arrived at a given diagnosis [59]. This lack of transparency can compromise trust, both among healthcare providers who need to rely on AI tools in their clinical workflows and among patients whose care is directly affected by these decisions [9–11, 43]. On the legal front, frameworks for determining liability in AI-driven diagnoses remain largely undeveloped in LMICs. Moreover, the ethical sourcing of training data remains a fundamental issue. Recent evidence shows that standard convolutional networks can infer patient race from chest radiographs with > 0.90 AUC, yet follow-up work indicates that acquisition parameters and site-specific technical factors, rather than race itself, drive much of the downstream performance disparity [60, 61]. Mitigation, therefore, requires both balanced datasets and harmonized imaging protocols.

One notable success story comes from the Colombian Association of Radiology, which has adeptly navigated legislative and policy challenges in integrating AI into medical radiology [62]. In 2019, the American College of Radiology (ACR) established an Artificial Intelligence Committee composed of experts in ethics and AI, effectively shaping Colombia’s regulatory framework for AI in healthcare. Their contributions to national white papers on AI ethics and responsible use aligned AI adoption with the government’s broader digital transformation agenda, ensuring top-down support and smooth implementation [63].

Crucially, the ACR partnered closely with Colombia’s Ministry of Health and other governmental bodies to address policy gaps and set clear guidelines for AI usage—covering areas such as data privacy, patient autonomy, and informed consent protocols [64, 65]. Through active participation in national forums and policy discussions, the ACR cultivated a regulatory environment balancing innovation with ethical responsibility [63]. This example highlights how collaborative efforts between professional associations, government agencies, and international partners can forge equitable, sustainable AI integration—even in resource-constrained contexts.

### Financial and sustainability constraints

Finally, the high cost of setting up and maintaining AI-ready imaging infrastructure often proves prohibitive for LMICs, especially when competing priorities—such as infectious disease control—dominate healthcare budgets. Many advanced radiology tools, including MRI and CT scanners, carry significant upfront and ongoing operational expenses, making investment in AI a complex proposition for governments and public health systems [26, 27].

To mitigate these financial barriers, alternatives like low-field MRI (≤ 1.5 T) can offer cost-effective imaging solutions without sacrificing essential diagnostic capabilities [66, 67]. Additionally, the long-term success of AI implementation in LMICs hinges on reliable, ongoing funding. Public–private partnerships (PPPs) and the incorporation of AI priorities into global health financing frameworks (e.g., the World Health Organization’s (WHO's) funding initiatives) can help secure sustained investment and reduce the likelihood of projects stalling after initial pilot phases. By embedding AI as a core strategic priority within LMIC healthcare systems, policymakers and stakeholders can foster durable solutions that continue to evolve alongside technological advancements.

## Strategic recommendations

Achieving effective, sustainable AI integration in LMIC healthcare systems requires a deliberate and multifaceted approach. The following recommendations focus on practical solutions, drawing on the challenges previously identified, to ensure AI can be adapted to the realities of resource-limited settings without repeating the entire challenge narrative.

### AI infrastructure and accessibility

Governments and healthcare institutions should prioritize cost-effective, portable imaging solutions (e.g., handheld echocardiography) to expand diagnostic reach and ensure equitable access to lifesaving services [20, 21]. Additionally, low-field MRI scanners can lower purchase and maintenance costs, making them viable for smaller clinics and rural hospitals [26, 27].

**Fig. 3:**
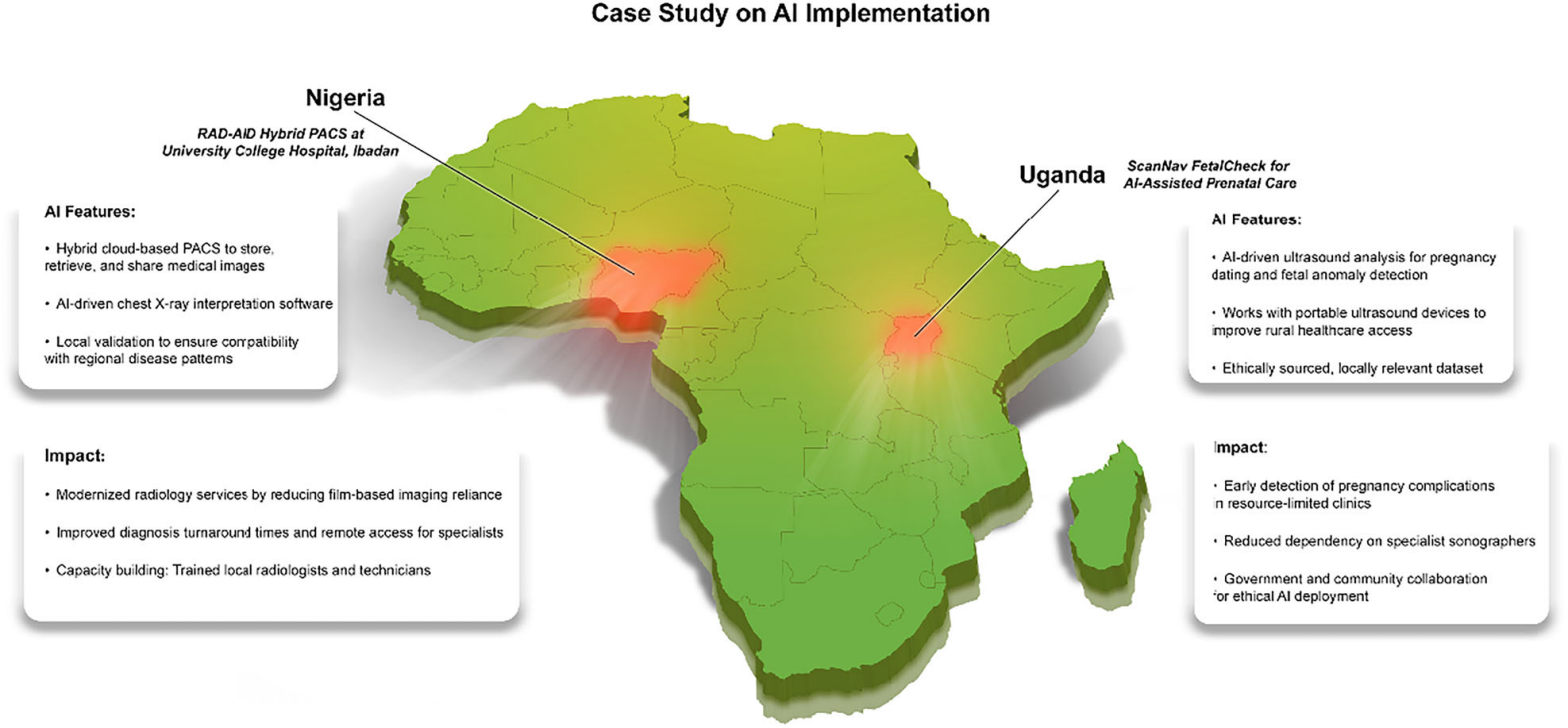
Case study on AI implementation (Nigeria and Uganda)

Where possible, healthcare systems and Non-Governmental Organizations (NGOs) should replicate or adapt successful models like RAD-AID’s hybrid cloud-based PACS in Nigeria [17]. Such strategies combine infrastructure upgrades with locally tailored AI deployments, reducing dependency on external expertise and empowering local professionals to operate and maintain AI-driven imaging systems. Figure 3 shows case studies on AI implementation in Africa.

### Data and AI model development

Rather than dwelling on data scarcity challenges, the focus here is on practical approaches to building robust, ethically sourced, population-specific datasets. Projects like ScanNav FetalCheck in Uganda demonstrate how transparent community engagement, privacy safeguards, and collaboration with government stakeholders can build trust and ensure sustainability [28, 68].

Researchers and policymakers should promote bespoke or fine-tuned AI models—like SAMBA for glioma segmentation—to accommodate regional disease profiles, imaging constraints, and underrepresented demographics [12, 13]. Combined with open data initiatives (e.g., AfNiA), these methods mitigate the risk of exacerbating health disparities and emphasize local validation instead of relying solely on HIC-trained models.

Recent advances in synthetic health data offer a promising, cost-effective solution to the scarcity of population-specific datasets. By generating AI-driven simulated datasets that preserve statistical properties of real-world data without exposing patient identities, synthetic health data can supplement existing datasets and help improve model generalizability across diverse populations [69]. This emerging approach merits further exploration as a complementary strategy for AI model development in LMICs, particularly where real-world data access is constrained due to privacy, regulatory, or logistical barriers.

### Workforce and training expansion

Cutting-edge AI technology alone is insufficient without a cadre of skilled professionals to implement and oversee it. Integrating AI education into medical school curricula, radiology residency programs, and continuing professional development courses ensures that clinicians become fluent in both AI fundamentals and practical imaging workflows [48, 49].

Train-the-trainer models (e.g., SWiM and CONNExIN) help create self-sustaining pipelines of AI experts who can mentor new adopters, troubleshoot local challenges, and tailor external models to local needs. Beyond technical skill-building, retention policies addressing brain drain—such as career advancement pathways and collaborative research opportunities—can keep professionals invested in their home regions.

### Ethical and regulatory reforms

Robust governance is paramount for clinician and public trust in AI-based diagnostics [70]. LMIC governments can establish or empower AI regulatory bodies modeled on success stories like Colombia’s AI Committee, ensuring transparency, patient autonomy, and data privacy. Early engagement with professional organizations helps align new regulations with national health priorities, creating an environment that balances innovation with ethical responsibility [9–11, 43].

### Financing and sustainability strategies

Long-term financial planning is essential for sustaining AI initiatives beyond initial pilot phases. Policymakers and international health organizations should prioritize AI in global health agendas—integrating AI-driven solutions into disease control strategies and broader healthcare investment plans [71–73]. In addition, PPPS can leverage private-sector innovation and public-sector infrastructure to create cost-effective, scalable AI deployment models capable of thriving in resource-constrained environments.

### Science diplomacy and collaborative partnerships

Science diplomacy serves as a powerful catalyst for bridging resource gaps and accelerating AI innovation in LMICs. International forums, cross-border academic alliances, and intergovernmental initiatives encourage technology transfer, shared research agendas, and mutual capacity-building. These diplomatic channels also foster policy alignment—ensuring that AI frameworks developed in one country can be adapted and ethically deployed in another. By championing transparent communication and global collaboration, science diplomacy not only enhances research impact but also ensures that AI adoption remains equitable, context-specific, and mutually beneficial to all stakeholders involved.

In addition to government and academic partnerships, public–private collaborations play a critical role in advancing AI-driven healthcare solutions. Tech companies, NGOs, and healthcare institutions are increasingly working together to develop and scale AI innovations that address public health challenges in LMICs. For example, computer-aided TB screening is operational at a national scale via platforms such as CAD4TB, which analyzed > 1 million chest X-rays across 14 countries with area-under-curve values of 0.86–0.91 [74]. This demonstrates how industry expertise can be leveraged to enhance public health initiatives [75]. By combining corporate AI capabilities with local clinical expertise, such collaborations accelerate deployment, ensure real-world validation, and facilitate scalability across diverse settings.

Expanding structured PPPs in LMICs can support AI training programs, data-sharing initiatives, and affordable technology deployment. When aligned with ethical guidelines and local healthcare priorities, these collaborations foster sustainable AI integration and prevent dependency on external expertise. Moving forward, multi-sector engagement will be key to ensuring that AI solutions are not only technologically sophisticated but also socially and culturally attuned to the healthcare needs of LMIC populations.

### Structural equity and epistemic justice

Beyond practical barriers, AI roll-out is shaped by deeper structural forces. Digital dependency describes a scenario in which LMICs supply data but must purchase finished algorithms from high-income vendors; data extractivism frames this imbalance as a twenty-first-century resource trade [6]. Participatory design and decolonial AI movements, therefore, call for communities to co-create tools that reflect local diagnostic priorities, languages, and risk tolerances [7]. Stakeholders could operationalize these principles by pairing every technical pilot with a governance compact that specifies data ownership, algorithmic audit rights, and benefit-sharing mechanisms—steps already pioneered by Kenya’s National eHealth Strategy and Peru’s open-data decree [8]. Embedding such compacts early will enhance the legitimacy and long-term sustainability of the technical recommendations described above.

When combined, the levers outlined above can shorten diagnostic waiting times by up to 60% (low-field MRI pilots in Nigeria) and raise tuberculosis (TB) case-detection in peripheral clinics by 20% (AI triage in Uganda), ultimately reducing morbidity through earlier treatment initiation and more efficient referral pathways. We highlight these numbers to make explicit the patient-level impact that motivated our roadmap.

## Implementation strategies

**Fig. 4:**
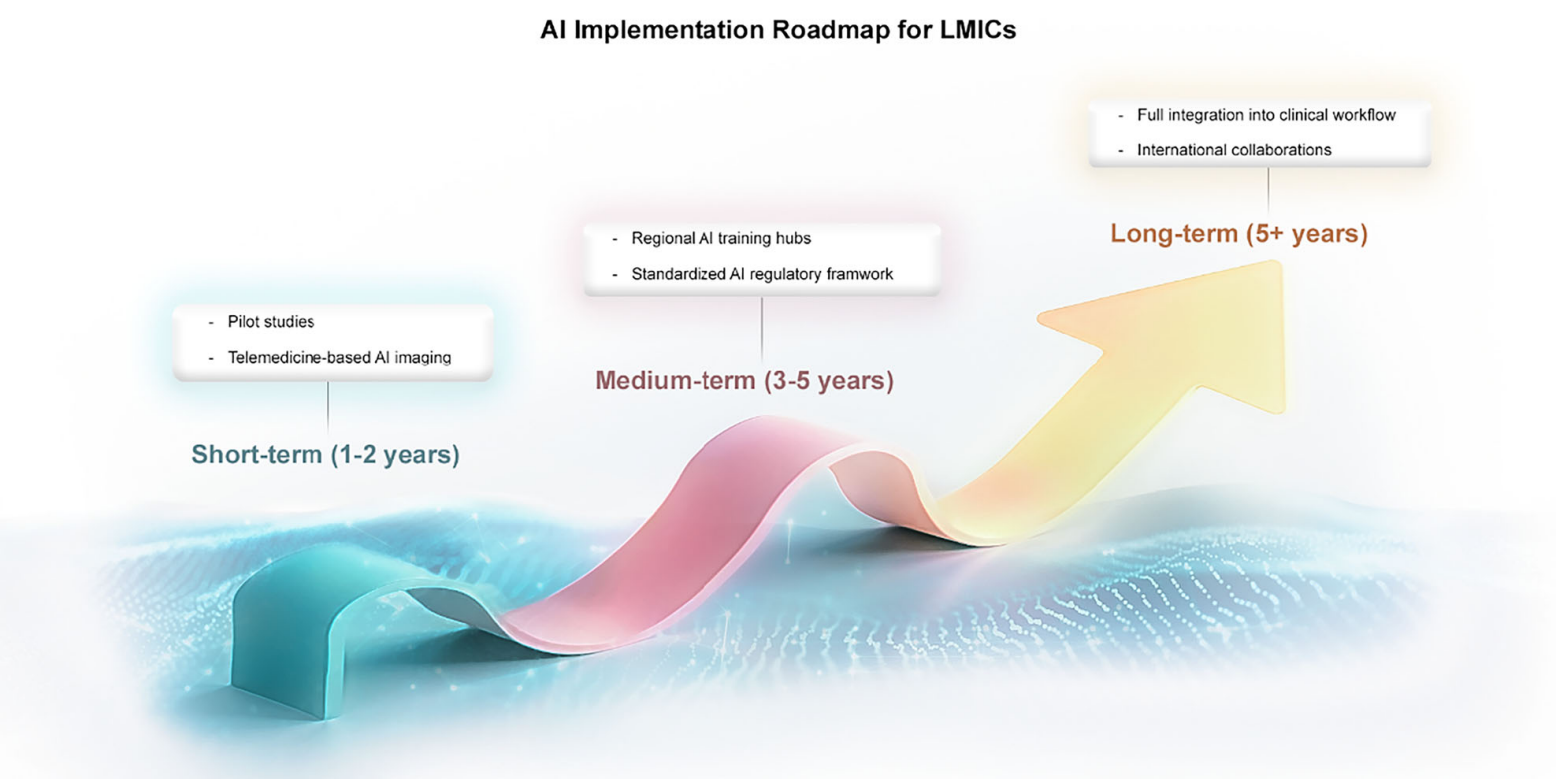
AI Implementation Roadmap for LMICs

The timeline for implementing AI-driven imaging solutions can be divided into overlapping phases—short-term, medium-term, and long-term—each with distinct priorities and objectives (Fig. 4).

### Short-term (1–2 years)

In the immediate stage, pilot programs focusing on AI-assisted diagnostic tools should be introduced in selected LMIC hospitals. By limiting the scope to a few institutions, policymakers and clinicians can carefully observe how AI systems perform, gather valuable feedback, and refine integration protocols before broader deployment [21, 23]. Telemedicine-based AI imaging platforms can also be expanded to underserved regions, where local expertise might be minimal, but connectivity may suffice for remote consultation and support. These initial steps allow for a controlled, measurable rollout of AI solutions and set the stage for more extensive adoption in subsequent phases.

### Medium-term (3–5 years)

Building on pilot successes, the medium-term strategy involves creating regional AI research and training hubs to foster sustained capacity building. These hubs can serve as centers of excellence, offering advanced training for healthcare professionals, facilitating collaborative research, and sharing best practices among neighboring countries [48, 49]. Alongside these capacity-building efforts, policymakers should seek to implement standardized AI regulatory frameworks in select LMICs, ensuring that legal guidelines and accountability measures keep pace with accelerating technological change [9–11, 43]. This period is pivotal for embedding AI more deeply into national healthcare systems while maintaining a structured and ethically informed approach.

### Long-term (5+ years)

Ultimately, the goal is to fully integrate AI-assisted medical imaging into the broader healthcare fabric of LMICs. By this stage, the foundation of robust infrastructure, local expertise, and well-defined regulations will support the seamless use of AI in everyday diagnostic workflows [71–73]. Cross-border collaborations become increasingly vital in advancing both innovation and equity, as countries with more established AI capacities can share knowledge, resources, and lessons learned with those still ramping up. Sustained partnerships, regional initiatives, and global research consortia will help maintain momentum, ensuring that AI’s transformative potential continues to be leveraged for the benefit of patients everywhere.

## Limitations and future research

Looking ahead, ongoing research and innovation will be key to maximizing the benefits of AI for LMIC healthcare. Longitudinal studies examining AI’s long-term impact on health outcomes, diagnostic accuracy, and cost-effectiveness are critical for evidence-based decision-making. Additionally, AI-driven clinical trials tailored to region-specific diseases—such as malaria, TB, or certain cancers prevalent in tropical regions—can generate insights into how models perform under real-world conditions and guide targeted improvements. Finally, as sustainability and environmental concerns gain prominence, research on climate-friendly AI applications will help ensure that future radiology practices minimize their carbon footprint while maximizing patient care. By pursuing these directions, stakeholders can forge a path toward more adaptable, equitable, and enduring AI-driven healthcare solutions in LMICs.

### Study limitations

Our synthesis is constrained by a single 2-h workshop and an English-language scoping review; francophone and lusophone literature was largely missed. Health-system shocks after February 2025 (e.g., Ghana’s new AI-in-Health Act) could already be reshaping the landscape. Finally, we did not empirically test the participatory-design frameworks discussed above; future prospective studies should assess their effect on algorithmic generalizability and community trust.

## Conclusion

AI implementation in medical imaging for LMICs holds immense potential to transform healthcare outcomes, but this journey is fraught with challenges spanning infrastructure, data, workforce, and ethics. The strategic recommendations outlined in this document—ranging from building robust regulatory frameworks to investing in local capacity and developing population-specific datasets—provide a roadmap for equitable and sustainable AI adoption. Yet, the path ahead requires dedicated collaboration among governments, medical associations, research institutions, and international partners.

As we reflect on the key themes of this consensus, four central pillars emerge as critical to the future of AI in LMIC radiology. First, ethical foundations are paramount; initiatives like AfNiA and the ACR work in Colombia demonstrate that ethical data acquisition, privacy protections, and alignment with local healthcare needs foster trust and maximize impact. Second, workforce empowerment is non-negotiable. Programs such as SWiM and CONNExIN show how targeted training and capacity-building equip local professionals to harness AI effectively, mitigating concerns like brain drain. Third, policy innovation—exemplified by the ACR’s legislative and regulatory successes—underscores the importance of collaboration between medical associations, governments, and international stakeholders in creating enabling environments for AI. Finally, collaborative models are key to sustainability; whether through partnerships like the BraTS Challenge or regional networks like CAMERA, cooperation ensures that AI adoption remains inclusive, equitable, and impactful.

Taken together, these pillars reaffirm that the integration of AI into radiology in LMICs is more than a technical challenge—it is also a moral imperative. By prioritizing ethical practices, investing in local capacity, and building inclusive policy frameworks, we can ensure that AI becomes a catalyst for global health equity rather than a tool of exclusion. The insights and best practices compiled here offer a guiding framework for stakeholders committed to leveraging AI’s transformative potential, while safeguarding the dignity and well-being of communities worldwide.

## Abbreviations

ACR: American College of Radiology
AfNiA: Africa neuroimaging archive
AI: Artificial Intelligence
CAMERA: Consortium for Advancement of MRI Education and Research in Africa
CONNExIN: Comprehensive neuroimaging analysis experience in resource-constrained settings
CT: Computed tomography
FAIR: Findability, accessibility, interoperability, and reusability
HICs: High-income countries
LMICs: Low- and middle-income countries
MRI: Magnetic resonance imaging
NGO: Non-Governmental Organization
PACS: Picture archiving and communication system
PPPs: Public–private partnerships
SAMBA: Segment anything model with bounding-box-guided prompts and an ensemble voting network
SWiM: Scan with me
TB: Tuberculosis
WHO: World Health Organization
